# Comparing Clean Water Act Section 316(b) Policy Options

**DOI:** 10.1100/tsw.2002.176

**Published:** 2002-02-19

**Authors:** John Kadvany

**Affiliations:** Policy and Decision Science, 1070 College Avenue, Menlo Park, CA 94025, USA

**Keywords:** adverse environmental impact, aquatic ecology, Clean Water Act, coolingwater intake systems, decision analysis, entrainment, environmental policy, fish populations, fisheries, impingement, multiple values, power plants, regulatory affairs, risk, Riverkeeper, stakeholders, tradeoffs, U.S. Environmental Protection Agency, Utility Water Act Group

## Abstract

This paper develops a comparative framework for policy proposals involving fish protection and Section 316(b) of the Clean Water Act (CWA). Section 316(b) addresses the impingement and entrainment of fish by cooling-water intake structures used principally by steam electric power plants. The framework is motivated by examining the role of adverse environmental impacts (AEIs) in the context of Section 316(b) decision making. AEI is mentioned in Section 316(b), but not defined. While various AEI options have been proposed over the years, none has been formalized through environmental regulations nor universally accepted. Using a multiple values approach from decision analysis, AEIs are characterized as measurement criteria for ecological impacts. Criteria for evaluating AEI options are identified, including modeling and assessment issues, the characterization of ecological value, regulatory implementation, and the treatment of uncertainty. Motivated by the difficulties in defining AEI once and for all, a framework is introduced to compare options for 316(b) decision making. Three simplified policy options are considered, each with a different implicit or explicit AEI approach: (1) a technology-driven rule based on a strict reading of the 316(b) regulatory text, and for which any impingement and entrainment count as AEI, (2) a complementary, open-ended risk-assessment process for estimating population effects with AEI characterized on a site-specific basis, and (3) an intermediate position based on proxy measures such as specially constructed definitions of littoral zone, sensitive habitat, or water body type. The first two proposals correspond roughly to responses provided, respectively, by the Riverkeeper environmental organization and the Utility Water Act Group to the U.S. Environmental Protection Agency (EPA)’s proposed 316(b) new facilities rule of August 2000; the third example is a simplified form of the EPA’s proposed August 2000 new facilities rule itself. The simplified policy positions are compared using the three dimensions of the comparative policy framework: (1) the role of CWA philosophy or vision, such as the use of technology-forcing rules, (2) regulatory policy implementation, and (3) the role for scientific information and the knowledge base. Strengths and weaknesses of all three 316(b) policy approaches are identified. The U.S. EPA’s final new facilities rule of November 2001 is briefly characterized using the comparative policy framework and used to further illustrate the approach.

### CLEAN WATER ACT SECTION 316(B) AND DEFINING ADVERSE ENVIRONMENTAL IMPACT

Section 316(b) of the Clean Water Act (CWA) is a remarkably brief and controversial piece of environmental law regulating cooling-water intake structures (CWIS). A CWIS is the structure used by steam-electric power plants (and some manufacturing facilities) to obtain water from a nearby river, lake, ocean, or estuary to cool purified steamwater that rotates the turbines generating electricity. Recooled steamwater is recirculated, while the cooling water from a "once-through" cooling system is returned to the source water body. Millions or even billions of gallons of water per day may be conveyed through a CWIS in this way. Consequently, adult or juvenile fish, or fish eggs and larvae, may be injured or killed, either by being *impinged* on external screens or other barriers, or by being *entrained* by the cooling water as it passes through the cooling system proper. Actual mortality or injury is not always 100%, and can depend on factors such as temperature gradients, species-specific survival strength, CWIS and cooling system design, intake flow velocities and gradients around the CWIS, types of protective mechanisms, and water body characteristics.[1]

Section 316(b) is intended to protect fish (or fish populations) from the potential hazards created by a CWIS. In full, Section 316(b) states: "Any standard established pursuant to section 301 [regulating effluent limitations] or section 306 [describing effluent performance standards] of this Act and applicable to a point source shall require that the location, design, construction, and capacity of cooling water intake structures reflect the best technology available [BTA] for minimizing adverse environmental impact [AEI]."

Does the key 316(b) term "adverse environmental impact" (AEI) need to be defined explicitly to advance 316(b) policy? The CWA provides little or no direct guidance on what AEI should mean, and no further explicit characterization has been provided to date by the U.S. Environmental Protection agency (EPA). Advocates on different sides of the 316(b) debate hold a variety of views. The electric power industry, at least as represented by its advocacy group the Utility Water Act Group (UWAG), and the Electric Power Research Institute (EPRI), have said that, without a characterization of AEI, it is not possible to know what impact is to be minimized and therefore how to select BTA. They have both also asserted that AEI should at the least only refer to the health and fecundity of fish populations as a whole, and not the acute impacts caused directly by impingement or entrainment. The Riverkeeper environmental organization, which successfully brought suit against the EPA in 1995, after the EPA failed for decades to promulgate 316(b) regulations, takes AEI to include *any* mortality to fish or other aquatic life caused by a CWIS. They also consider "closed-cycle" dry cooling to be BTA.[2] (A "dry" closed-cycle system is like a radiator in which evaporative loss is minimized, while a wet closed-cycle system, such as a cooling pond or open tower, allows evaporative loss. Both systems recirculate cooling water instead of returning it directly to its source. An evaporative system may count as a consumptive water use, unlike a once-through system, and hence may be subject to regulatory constraints governing in-stream flows.)

The Riverkeeper position may be supported by a straightforward (but perhaps overly-literal) reading of the short 316(b) text, notwithstanding the ambiguity associated with AEI and BTA. But a consequence could be the retrofitting of many existing CWISs at high costs (in the tens to hundreds of millions of dollars per plant) for the industry. For new facilities, a strict 316(b) reading could imply using cooling alternatives, such as recycling cooling towers, that have other environmental impacts. UWAG and others contend that the ecological benefits that would be obtained by eliminating an existing CWIS or using closed-cycle cooling are almost always, if not always, minimal or zero. That while thousands or tens to hundreds of thousands of individual fish may be killed yearly by a single CWIS, and even billions or trillions of fish eggs and larvae, such mortality usually has no important impact on a fish population as a whole, especially in the context of commercial and recreational fishing, which has a far greater influence on fish population size and health. Regardless of technology cost, the electric power industry also contrasts the Riverkeeper position with numerous examples of natural resource management (fisheries, forests, and livestock) for which only populations are relevant.

In any case, no existing CWIS has ever been replaced (though many have been modified) because of 316(b). While regulatory motivations are a matter of conjecture, the reasons likely include a combination of the high cost of retrofitting (cooling system design is fundamental to plant efficiency and hence cannot easily be compensated for if changed); the near impossibility of eliminating *entrainment* in once-through systems (by contrast, impingement, while often challenging to control, is more manageable and less costly); and, most of all, the difficulty in establishing that the ecological disturbance change by a CWIS indeed constitutes ecological harm. The Riverkeeper asserts that such large numerical impacts are of obvious ecological significance, while the industry considers that judgment to be a superficial risk perception at odds with fisheries science and the management of fish resources generally. Possible adverse environmental outcomes, Section 316(b)'s "AEI", therefore are part of a complicated decision often involving unique ecological circumstances, a shortage of useful remediation options, and unclear policy about ecological value. The question of AEI is in part a scientific judgment regarding just what happens to fish in the water. It is also a nonscientific value judgment regarding whether an ecological change is to be deemed adverse, while 316(b) language gives no particular guidance about the adverse impacts to be "minimized."

Hence it is reasonable to return to the 316(b) text and ask: "What is an AEI"? Why could the EPA not create a substantive and workable AEI definition (not to mention actual regulations) over the decades since the CWA was enacted and the suit brought by the Riverkeeper? Without providing details of this fascinating and important story in environmental regulation, and with due respect to the Riverkeeper's position that any form of CWIS-induced fish mortality constitutes AEI, the following contrasts should suggest sources for the controversy and the EPA's difficulties in grappling with CWIS decisions:

- Fish are a consumed natural resource *and* an ecological resource to be protected.
- The large numbers often associated with fish impingement and entrainment may be of *prima facie* concern *and* of little evident concern from the perspective of fisheries management, while EPA's regulatory scope does not include fisheries management per se.
- Judgments of ecological quality or health depend on science for their prediction *and* an implicit or explicit social value judgment for their importance.
- Fish are valued differently when they are created by a stocking program in a reservoir, or are essential to the productivity of a precious estuary, or are a nuisance fish to be eliminated, or are so fecund that huge croppings may be accepted with no concern.
- While risk assessment does not distinguish fish mortality caused by fisherman or other predators from mortality caused by a CWIS, the inconsistency, if there is one, of so doing can nonetheless be upheld by a society and codified as law.
- The ecological changes caused by impingement or entrainment can be hard and costly to predict with few or no generalizable indicators across a variety of water body types(e.g. rivers, estuaries, oceans, lakes and reservoirs), and with the causal importance of various anthropogenic and nonanthropogenic influences also being hard to disentangle.

Defining AEI, therefore, has embedded in it several factors contributing to making 316(b) risk controversial and resistant to obvious risk management solutions. It is an important example because it primarily involves ecological and not human health risk where such controversies most often arise.

UWAG has recently proposed an AEI definition, but it amounts to site-specific risk assessment of fish population impacts due to an existing or future CWIS.[3] The definition is broad enough to include any possible stakeholder concern, but provides no specific criteria for levels of acceptable population decrease; that choice would be part of a site-specific risk and values assessment. It is a reasonable guess that UWAG arrived at their proposal for site-specific risk assessment after recognizing that useful and meaningful general rules (e.g., "*x%*

reduction in regional population size" or "probability of at least *p* that population falls below critical level *x* for *n* years") for characterizing adverse ecological impacts are almost impossible to define in the CWIS context. The industry, seeing also that dozens of 316(b) decisions have been made over the years through the National Pollutant Discharge Elimination System (NPDES) permitting process *without* detailed regulatory guidance, also feel confident proposing a more flexible process in which AEI is effectively defined locally by regulators and stakeholders. The only real constraint on that would be limiting ecological impacts to the population level (except for species covered by the Endangered Species Act).

Thus the Riverkeeper and UWAG have staked out polar positions on AEI, with the EPA still not opting to define AEI. Instead, the EPA proposed in August 2000 to regulate new (vs. existing) CWISs by water body type (estuary, river, marine, lake) along with a set of supporting proxy measures, such as location of CWIS relative to a defined "littoral zone," and CWIS intake flow velocity. These proxy measures are intended, one presumes, to protect aquatic life from AEIs, though the latter is again not characterized. A rough summary of the positions staked out by the Riverkeeper and UWAG in response to the EPA's August 2000 proposed new facilities rule is contained in the Appendix, and similar issues may be raised through debate over existing facilities rules. For the purposes of this paper, a policy goal is assumed of some intermediate position between an open-ended site-specific risk assessment and stakeholder process, like UWAG's, and the Riverkeeper's strict reading of the brief 316(b) text. Just what to take and reject from these is part of the policy decision faced by the EPA. This policy goal is assumed here as a means of exploring the main proposals put forward, and because the 316(b) policies finally adopted by the EPA may be the result of multiple compromises or policy tradeoffs.

To compare 316(b) policy options, the paper begins by asking whether defining AEI is a good starting point for organizing the environmental, stakeholder, scientific, and regulatory issues involved in articulating a coherent 316(b) policy. The answers provided are Yes and No. Yes, because the problem of characterizing AEI leads to a broader set issues which should be addressed by any defensible 316(b) policy; No, because these issues cannot be answered only through an *a priori* or general AEI definition. AEI is important because it stands for environmental and stakeholder consequences or outcomes generally; thus it is a necessary piece of environmental regulation. However, it is not sufficient, and much of the 316(b) controversy can be understood by looking for other institutional, regulatory, and judgmental factors underlying 316(b) policy design. It is important to understand why this approach is being taken. From a broad values-based stakeholder perspective, in which 316(b) decisions incorporate whatever ecological, social, and financial are considered relevant, AEI should just reflect appropriate local or societal value judgments about impacts on biological health. Indeed, the position of many in the electric power industry has been close to simply taking 316(b) to be a site-specific risk assessment and decision process that allows just that. What should be made of opposing views to what is arguably a widely held approach to environmental decisions? What are rationales, if any, for their alternative views? The goal here is not to defend any particular view, but to understand the logic behind some complicated combinations of policy choices.

This paper's approach, therefore, is to show how evaluating pros and cons of AEI options leads to other important 316(b) policy choices for regulators and stakeholders. These additional questions about 316(b) policy arise regardless of whether a definition of AEI is central to, or explicit in, a 316(b) policy proposal. The questions define the policy space, so to speak, in which various 316(b) policies can be defined. EPA's first attempt, in 2000, to forge 316(b) policy for new CWISs through synthetic proxy definitions can be seen as a classic institutional response to the messy but tractable reality of an environmental decision burdened by poor regulatory history, considerable stakeholder interests, and unclear scientific and social directives. The Riverkeeper's and UWAG's options are characterized as strong on some dimensions, but weak on others. Different options that build on these three positions are possible. The reader can decide which, if any, are desirable, including the EPA's November 2001 final new facilities rule[4]. In any case, one goal of this paper is to show that comparing existing 316(b) policy options, and defining new ones, can be simplified and made considerably more transparent. By starting with the problem of defining AEI, challenges for risk assessment and public policy are raised by two roles for the Clean Water Act: as enabling legislation for 316(b) regulation and as law that has to be practically implemented with respect to substantive but imperfect science and competing stakeholder values.

The next section first locates AEI in the context of 316(b) decision making. That perspective will suggest other policy questions whose answers help evaluate the merits of AEI proposals. Tradeoffs in satisfying all 316(b) policy needs are raised, and these tradeoffs are used to define comparisons for 316(b) policy options.

### WHAT IS AN AEI?

What is an AEI generically thought of as a component of environmental decision making? That is, what role does AEI play in actual 316(b) choices, analogous to other environmental decisions? The answer is that an AEI is a measure or criterion for regulators and stakeholders to evaluate the ecological or other benefits and costs of making 316(b) choices. Examples may include:

- Any acute mortality to adult fish, larvae, or eggs; similar acute mortality, but above some fraction of the total species population size, or above some absolute number;
- Acute mortality to adult fish only;
- A decrease in fish population size threatening its long-term local or regional viability, but not acute impacts per se;

- A probability of fish population decline greater than some critical value;
- A similar probability of decline for multiple species;
- Any of the previous, but ignoring invasive or nuisance species;
- Estimated economic impacts to commercial fisheries and recreational losses;
- Ecological productivity losses to aquatic populations beyond those immediately affected by the CWIS.

316(b) *choices* here means selecting among impingement or entrainment reduction technologies or approaches of any kind, including barrier or diversion devices, CWIS operational, location, or design changes, use of dry or wet closed-cycle cooling; and, for completeness, mitigation or enhancement options, such as fish stocking or ecological restoration projects. The latter may be chosen if it is decided that CWIS modification itself has low benefits, but that a compensating action should be taken nonetheless (as may occur when CWIS technology costs are extremely high). AEI measures may be taken to include any required definitions (e.g. fish population geographical ranges, relevant species) involving ecological scale and biological function (e.g., reproductive success, predator-prey relations, energy transfers), and the judgmental, empirical, and mathematical means used to make such definitions operational. AEI for practical purposes includes *how* AEI is determined as well as *what* it is or should be.

This characterization of AEI follows from identifying generic categories of stakeholder *objectives and values* associated with 316(b) decisions as a whole. These stakeholder values include:

- The ecological consequences to fish and other aquatic (or even terrestrial[4]) species, at any level of ecological scale;
- The direct capital and operating marginal cost of the technology choice (e.g., barrier installation and maintenance, flow reductions, intake relocation, etc.);
- Energy production changes associated with a 316(b) choice, e.g. comparative efficiency losses due to cooling towers;
- Economic impacts on relevant commercial fisheries;
- Changes to recreational fishing, possibly including a mix of noneconomic and economic factors;
- Land use or aesthetic issues associated with the use of cooling ponds or towers;
- Ecological changes associated with mitigation options possibly chosen in lieu of impingement or entrainment reduction; and
- Possible environmental side-effects such as water quantity use and changed air emissions, due for example to the use of cooling towers in place of a CWIS.

Figure 1 graphically summarizes these 316(b) stakeholder values. Benefits and costs here have their broadest possible meaning, and are not limited to market-valued resources.[5]

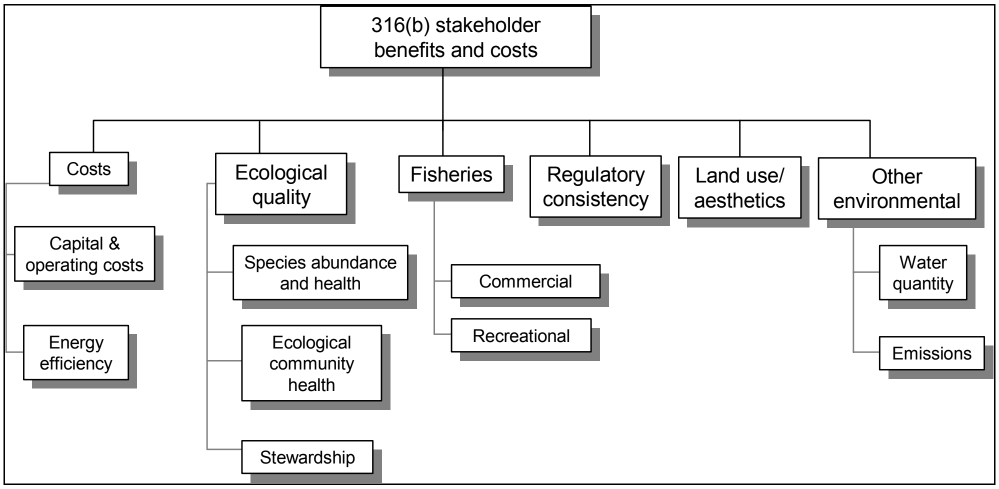

**FIGURE 1.** Stakeholder values relevant to most 316(b) decisions. Graphical organization does not indicate any priority. Measurement criteria are required for all categories and may differ for different 316(b) decisions.

Whether defined by the CWA or elsewhere, these are value categories that are relevant to 316(b) decisions. In particular 316(b) decisions, different subsets may assume greater or less importance and their measures may be operationalized differently. But however a 316(b) decision is actually made, it implies changes for some of these stakeholder values. These changes are measured somehow, and the measures associated with biological or ecological change effectively define AEI, or its absence, for that decision.[6] For example, suppose a 316(b) choice is to install a barrier net to reduce impingement and to carry out a fish stocking program to mitigate entrainment. That choice might be labeled the "best technology available" (BTA) selection, as provided by the 316(b) text. But its benefits and costs depend on ecological quality changes; a fisheries benefit due to impingement reduction; plus benefit to be achieved through the stocking program (including the possible continuing negative effect, if any, of entrainment); and all technology and enhancement program costs. AEIs in this way are determined by the quantitative or qualitative measurement criteria used to evaluate stakeholder interests or make 316(b) choices, even if not as explicitly organized as in Fig. 1. That perspective applied even to choices assuming the most conservative technology standard, such as limiting BTA to dry cooling.[7] For even if a technology standard is not intended as a direct measure of environmental change, it implies changes in benefits and costs, and thus becomes a proxy measure in practice. In such a situation, regulators or stakeholders will effectively back-calculate, as best they can, the consequences of interest as shown in Fig. 1. Section 316(b)'s BTA language makes it sound like a technology-based standard, along the lines of much of the CWA. But in practice, BTA decisions, including broadly defined technology rules, incorporate measures effectively defining AEI. Thus, it matters in the end not whether a biological change is labeled "adverse."

Rather, what matters is simply what 316(b) choice (including no action), if any, is made, and how outcomes associated with that choice differ from the status quo or other benchmarks.

Thus we have a first organizing principle for AEIs. Starting with a values-focused view of 316(b) decisions, AEIs are implied by the many options available for measuring, and then comparing, the benefits and costs for stakeholder interests. This purely consequentialist perspective does not depend on limited or out-of-date views on economic cost-benefit, or the choices for how tradeoffs between competing values (e.g., dollar cost and ecological change) are carried out.[8] It is a ubiquitous feature of 316(b) decisions. So the problem of defining AEI, whether uniformly or on a site-specific basis, is first that of designing and implementing such measurement choices, and second, deciding which measured levels constitute "adverse." More generally, one may evaluate the tradeoffs accepted among various 316(b) choices for all relevant consequences or outcomes. The multiple values perspective of Fig. 1 shows that ecological AEI only is only one piece of the 316(b) decision-making process.

In principle, everything needed to compare a set of 316(b) choices for a new or existing CWIS is contained in Fig. 1 plus criteria for measuring each value category.[9] First, one or more proposed 316(b) entrainment or impingement reduction technologies, CWIS operational changes, mitigation proposals, or other options, are evaluated along the dimensions in Fig. 1. Then, regulators or stakeholders (implicitly or explicitly) rank or compare options based on their value judgments (e.g., comparative value of ecological or fisheries change and technology costs) and 316(b) regulatory policy constraining choices (e.g. whether mitigation is allowed, and then what kinds). Such evaluations may integrate uncertainty about various outcomes, some combination of impingement and entrainment counts, forecasts of fish population changes, cost estimates, and so on. There also may or may not be a formal process for how 316(b) options are compared and ranked. Nonetheless, once we back up from AEI to the implied stakeholder value hierarchy in Fig. 1 and see what measurement criteria and tools are actually used, AEI has been effectively defined at least on a case-specific basis.

So 316(b) decisions, like all environmental decisions, will always effectively use some notion of AEI, whether AEI has been formally defined or not, and whether or not in advance of an individual 316(b) decision. That sounds like defining AEI should then be central to 316(b) policy. As mentioned above, the electric power industry has effectively framed a general approach to site-specific tradeoffs as an AEI definition, with the only major constraint being to focus only on fish populations, not acute impingement and entrainment. But as is argued next, while that consequentialist approach (or even one also allowing acute impacts) is correct in principle, the many measurement or AEI options possible make it difficult to provide much substantive guidance without further goals for what 316(b) policy should achieve and how that is to be accomplished. The many AEI alternatives may in this way be faced with decision-making constraints. This paper's main thesis is that 316(b) policy options are largely determined by how these decision-making constraints are interpreted and addressed. In the language of decision analysis, 316(b) policy alternatives revolve around major disagreements of the 316(b) decision *frame*; it is the decision "dog" wagged by the AEI "tail."

### EVALUATING AEI OPTIONS

This paper began by asking how important defining AEI was for formulating 316(b) policy. That question can now be revised.

Are the stakeholder values of Fig. 1 plus a set of measures, or AEI proposed definitions, adequate for formulating a substantive 316(b) policy perspective? To answer that question means evaluating various AEI options, including the option of allowing site-specific approaches for different rivers, estuaries, lakes, or marine environments. That is, imagine all the ways that AEI, whether explicitly or not, can or has been defined in a 316(b) decision. How are the choices or implementations of measures in an actual decision to be compared? Following are some of the evaluative questions that can be raised, with no answer intended as right or best. Importantly, these are not abstract questions, but ones that have occurred in actual 316(b) decisions (and are cited in the notes). Thus, even if AEI is not formally defined, these or similar questions about AEI adequacy will appear anyway, and imply real choices to be made by regulators or stakeholders.

*What are the roles for individual fish, fish populations, and/or ecological community measures?* Which fish population or acute mortality changes are measured? What additional ecological or trophic level changes may be measured, and over what temporal and spatial scales? How do such outcomes change depending on 316(b) technology choices? How are these selected for the aquatic life in a given water body, and what is the role of current resource management practices?[10]

*What is the role of proxies?* Technology standards, such as CWIS intake velocity criteria or biocriteria (i.e., measures integrating a suite of relevant ecological measures and judgments), can be seen as proxies for outcomes of genuine interest. For example, an intake velocity is not valued per se; it is a stand-in for achieving some genuine environmental objective. How well or poorly have 316(b) decision-making objectives been measured, given the use of such proxy measures? Do they work well for some water bodies but not others, as has been argued for biocriteria?[11] Are they as useful as other measurement approaches?[12]

What modeling approaches, simplifications, background assumptions, calibration techniques, and data collection methods are to be used? Of the many approaches for modeling fish populations, which are used and why? What is the quality of data used to estimate model parameters? What methods are used and over what time period? What types of judgment are needed to calibrate a model and ensure that it gives realistic or credible results? What background assumptions such as projected future hydrological flows and temperature regimes are assumed? How does the scientific process of theory proposal and testing fit in to 316(b), given the challenges of characterizing local ecological change?[13] Even if technological standards are used as proxies for site-specific modeling, one nonetheless makes some ecological assumptions, and uses some data and some conceptual model of a water body and its aquatic life.[14]

*How are constructed measures used?* Constructed measures are those created or designed specifically for a particular decision, and not necessarily applicable to others. These occur throughout environmental decision making, and examples from 316(b) could include estimates of trophic level changes specific to a particular water body or possible changes associated with a site-specific mitigation or enhancement project. What professional judgments and modeling or empirical considerations lay behind such a measure?[15]

*How are priorities set for species of concern?* In some situations, there will be too many fish species to track all of them, and typically several species dominate a 316(b) decision. How are these selected and related to one another? What is judged when or if, say, just a single species of several is doing poorly?[16]

*How are model outputs aggregated, and uncertainty and causality addressed?* In using fish population forecasting models, a typical output could be a population size *trajectory*, including confidence intervals, over multiple years and for multiple species. How are such stochastic trajectories aggregated over time and compared for 316(b) decision making? How are modeling and data uncertainty distinguished from natural variability, and how are probabilistic modeling outputs interpreted? If necessary, how are CWIS causal influences disentangled from nonanthropogenic influences such as water temperature and water quality, or overfishing, as another potentially important anthropogenic cause?[17]

*How is ecological value defined?* By what means are ecological changes compared to other stakeholder values, including costs? What do such comparisons mean? Are they explicit or implicit in the 316(b) decision-making process? To what extent may they be compared to other environmental decisions?[18] Do water body characteristics play a role in determining ecological value, such as differences between productive estuaries, managed reservoirs, or impacted water bodies? What is the role for considering changes to fish populations as opposed to acute mortality?

*What kind of stakeholder and risk assessment process is followed?* To the extent that any choices are made for assessing fish protection benefits, or comparing various stakeholder values, what process is to be followed? For example, are the suggestions made in the EPA's Ecological Risk Assessment guidelines (as yet not recommended by EPA for 316(b) but supported, for example, by EPRI) followed, or other risk assessment and stakeholder paradigms?[19]

*How are predictive accuracy, environmental benefit, and regulatory effort balanced?* What, in practice, are realistic options for reducing uncertainty for understanding fish population impacts, with or without various 316(b) choices? Regulators and others have limited resources to expend on studying a 316(b) issue. How much effort should be expended on data collection and modeling tasks, given options for deciding with or without more information, and relative to ecological and other consequences, including costs, associated with a particular decision?[20]

What is characteristic of the 316(b) policy debate is that the variety of water body types and uses, fish populations, and resource management settings makes a great range of reasonable and realistic answers possible to these questions. Indeed, because permanent 316(b) regulations (as opposed to the original CWA legislation) were effectively never created, the historical implementation of 316(b) has in practice led to a variety of site-specific answers. It would indeed be extraordinarily complex to enumerate all options and relevant conditions associated with various water body types, impingement and entrainment scenarios, ecological settings, and so on. It is also doubtful that they can be easily sorted out by starting with the question of defining AEI. Nor is there a generic 316(b) decision or paradigm that appears representative of all.

There are two immediate answers in response to the complexity associated with subtle environmental effects, challenging assessments, and widely varying ecological significance. One is to avoid AEI implementation issues altogether through a simplifying, uniform technology standard. Another is site-specific risk assessment through which AEI implementation is carried out case-by-case. However, each approach still needs some consideration of how it would be implemented to achieve CWA goals.

This implies that some broader context than AEI measures alone (or AEI plus stakeholders and their multiple values) is needed to compare policy options for the 316(b) debate. The next section proposes one such approach.

### ORGANIZING AEI EVALUATION

The first step in understanding the role of AEIs in 316(b) decision making was to recognize that AEIs appear as implicit or explicit ecological measurement criteria for 316(b) stakeholder values.[21] In the previous section, key questions about AEI measurement were raised, with the observation that there appears to be no satisfactory generic approach to defining AEI once and for all (or at least one different from counting any acute impact as significant). Following are three categories for organizing these questions and characterizing the adequacy of possible AEI measures; these evaluative categories are implicit in any 316(b)

policy approach and are not answered only by the comparison of stakeholder benefits and costs as illustrated in Fig. 1:

1. What is 316(b) trying to achieve in the context of the CWA?
2. How is that to be accomplished in practice?
3. What knowledge base informs these goals?

Figure 2 graphically summarizes the approach, followed by further explanation of how the questions help compare 316(b) policy proposals. Answers to these questions help characterize the decision frame, or fundamental constraints and assumptions, through which a 316(b) decision may take place.

*Clean Water Act 316(b) Policy Goals and Vision.* AEI proposals are tools for implementing a vision for what 316(b) policy is intended to be and what, in broader social, political, and regulatory terms, CWA legislation is, or could be, intended to make happen. For example, should 316(b) be organized along the lines of technology standards, as are many CWA wastewater rules?[22] That can be construed narrowly as one of legal interpretation, but that is not the intention here. Rather, for 316(b), are technology standards of various kinds a good idea for achieving CWA goals? Is it appropriate to "force" strong technology standards to improve environmental quality, as was done for waste- and stormwater through the CWA? A positive answer would be supportive of the Riverkeeper's position, and implies that aggregate "errors" (e.g., overprotection) made through a uniform rule are small compared to overall benefit. A negative answer implies other justification is needed for uniform standards; alternatively, a negative answer could mean that no feasible technology standard is justified by the benefits associated with 316(b) outcomes.

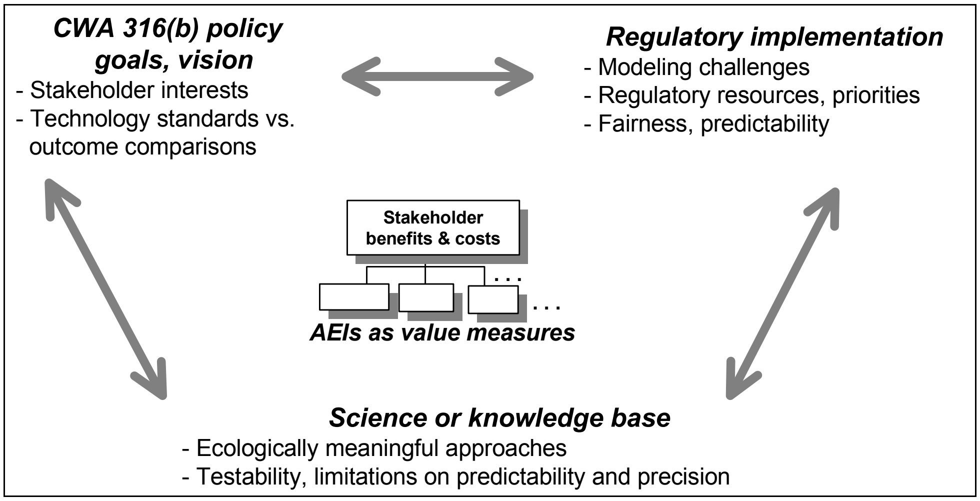

**FIGURE 2.** Comparative framework for evaluating AEI and 316(b) policy options.

Given that fish protection and harvesting is also managed and regulated outside of EPA, just what is EPA's role? One position is that "a fish is a fish," that a resource should be managed uniformly across all agencies. But that itself is a policy choice, so it is possible that one agency could decide that it has enabling powers not shared by others, and not regulate a resource in exactly the same way as others.[23] It may be that, for whatever reasons, we choose to protect a fish resource in one way and manage its consumption differently (e.g., because U.S. fisheries policy has many weaknesses), or we may not. Importantly, there are broad questions of equity and fairness between industries as consumers of the fish resource. That is, why should the electric power industry not be able to treat fish mortality as a resource harvest? Why should fish killed for food differ from fish killed to produce societally important electric power? It surely does not matter from the perspective of the fish.[24] These questions are not necessarily that difficult to answer, but some answer should be forthcoming. No obvious social values appear to decide the answer, as many people are likely unaware of the numbers of fish normally impinged and entrained as part of the power production process, and risk perceptions of how fish are killed may, for better or worse, significantly influence judgments of those outside the 316(b) debate. Framers of 316(b) language may not have anticipated the equity and resource consumption problem, either. Entrainment and impingement affect different phylogenetic levels, namely eggs and larvae vs. adult fish, about which societal values may be ambiguous, also. To make a stark comparison, if instead of fish, adult dogs or cats were killed, albeit with no significant population impacts, someone would surely take notice; whether that is "obviously" not true for fish appears unclear. This is not to argue that the Riverkeeper's position against almost all forms of fish mortality is correct, only that the basis for the ecological value choice is not transparent.

Other CWA issues include the role of technology costs, which are not mentioned in 316(b), but play a role elsewhere in the CWA. Relevant also to CWA philosophy is the role of cumulative risk in attaining the CWA goals of "swimmable, fishable" waters. Are 316(b) impacts to be regarded as individually small, but regulated as one of multiple aquatic hazards? The overall risk aversion or degree to which more certain or timely outcomes are preferred may also be a reason for supporting technology standards as opposed to allowing site-specific determinations.[25]

Finally, the BTA and AEI language of 316(b) likely would not be created today because of (at least for some) its evident ambiguity and brevity. The simplicity of 316(b) language is comparable in its logic to the now-repealed Delaney Clause of the Food and Drug Act prohibiting absolutely any amount of a carcinogen (e.g., from a pesticide used on a vegetable crop) to persist in processed food, regardless of the outcome to human health.[26] Similarly, 316(b) almost prima facie disallows any de minimus CWIS-caused impact, regardless of its ecological significance, and regardless of seeming inconsistencies with the management of other harvested resources. Much of the Riverkeeper's policy position depends on just such a strict reading. Therefore, it may be asked, though no major stakeholder group to date has done so, to what extent is a “rewrite” of 316(b) in order using more up-to-date concepts of risk assessment or stakeholder involvement? A negative answer implies sticking to traditional CWA approaches, while a positive answer implies consideration of broader risk management and stakeholder involvement options.

Various coherent 316(b) policy positions can be created by different answers to these CWA questions. The questions have in common that they should be addressed uniformly as part of 316(b) policy, and not reinvented locally when particular 316(b) decisions are made.

*Regulatory Implementation.* Implementation refers to the decision-making process required by regulators or resource managers, and involved stakeholders, to make 316(b) technology choices (including no action and mitigation); of particular importance is the *effort* needed to evaluate 316(b) choices compared to the *accuracy* of such assessments. .[27]

If modeling or empirical study are required, what kinds of requirements are useful for ensuring useful results? Both the Riverkeeper and the EPA have commented on the practical need to simplify 316(b) implementation, reacting in part to the challenges in assessing and modeling fish population changes due to a CWIS. However, mere technical difficulty is not a reason to impose a conservative technology standard. If it is judged that 316(b) problems generally do not pose especially significant ecological risks, then it is consistent to allow more leeway in local regulatory decisions, with the belief that the consequences of possible errors may be small. Conversely, if 316(b) represents a more important ecological concern, that may justify allocating or shifting more resources toward it and away from other water problems. But, if resources required for extensive study are great and leave uncertainty unresolved, then more uniform technology rules may be justified depending on their cost. Overall judgments of comparative risk and their tradeoffs thus are closely tied to regulatory implementation priorities.

In any case, regulators need to place 316(b) on their priority list for allocating their own and stakeholders’ resources. How important, for example, is 316(b) either as a watershed or fish protection issue compared to, say, nonpoint source runoff, combined storm-sewage water overflows, or other priority water quality and quantity issues? Or are regulators to set such priorities locally? More generally, what guidance is there on making ecological value judgments related to 316(b)?[28]

Finally, how predictable and fair is a 316(b) decision process, either for regulators or the regulated community? Are perceived fairness and predictability largely provided via technology standards? If technology standards are not in place, what expectations can the regulated community have for predictability and fairness of the decision-making process? For example, in historic 316(b) cases where technology options have been ruled out for cost or effectiveness reasons, mitigation projects have been allowed as a substitute, with costs ranging from hundreds of thousands to many tens of millions of dollars.[29] What fairness considerations are relevant here?

*Science or Knowledge Base.* Even given a CWA policy vision and an approach to how it should be implemented, any 316(b) policy or AEI proposal should relate *somehow* to knowledge and the science of fish lives, populations, ecosystems, and options for affecting ecological outcomes. For example, there are significant ecological differences between impingement of adult fish and the entrainment of eggs and larvae. The natural mortality rates of the latter can be above 90%, which, while not justifying entrainment, and depending on other causal influences, may mean that fish populations may be only weakly influenced by CWIS mortality at that life stage. That important biological difference in fish life stages also is not reflected in 316(b) language. Fish population declines should also account for other causes of fish mortality or population size changes including (over-) fishing, water quality degradation, and natural variability in water temperature and fish habitat. An important piece of policy guidance therefore is recognizing the influences on fish populations other than CWISs. Also important are the judgmental considerations relevant to assessing their importance, as it is often difficult to unambiguously model or assess causal strength for ecological changes associated with CWISs.

Policy guidance for 316(b) science can also be provided for different types of modeling, modeling outputs, data collection, and the simplifications often needed to calibrate and implement fish population models for practical use. In particular, if technology standards, biocriteria, or other regulatory proxies are to be used, then what are reasonable compromises intended for policy purposes? Finally, what is the role for fish science in valuing fish mortality at the individual versus population level, a major point of disagreement in the 316(b) debate? This is a value judgment and *not* a scientific question.[30] But since biologists are accustomed to making implicit value judgments about ecological health, policy advice is needed to distinguish neutral fish mortality outcomes from judgments of biological or social value. Biologists can participate in those judgments, but policy guidance is needed to distinguish, as best as can be done, roles for value-laden and technical judgments.

That completes the description of the three main areas of 316(b) policy evaluation. Most of these issues raised have been argued by others involved in the 316(b) debate. The organization provided here shows, first, that many questions that can be asked of 316(b) policy can be usefully arranged under the three broad categories described. Second, while any 316(b) policy implies an explicit or implicit role for AEI, one can ask many critical questions about how 316(b) policy could or should work without making an AEI definition axiomatic. That does not mean ignoring AEI, but putting it in the decision-making context created by the CWA, the need for pragmatic regulatory guidance, and the practical use of scientific knowledge.

Therefore these three evaluative dimensions, plus the values-focused perspective of Fig. 1, provide a simple functional model of 316(b) policy. AEI or their implied 316(b) policy proposals can be examined by seeing how well they perform along each dimension. What is important so far is that each policy component raises important issues, with no right or wrong choice in isolation, and whose answers should cohere with those provided from the other policy components. Any 316(b) approach needs to address each component to work well, or at least to be immune to some immediate objections.

### COMPARING SOME 316(B) POLICY STRATEGIES

Three simplified 316(b) policy strategies are now used to examine how the categories of the previous section (or Fig. 2) work in practice. While illustrative only, they indicate issues relevant to proposals from important 316(b) stakeholder groups.[31] The three simplified approaches are:

- *"Strict" 316(b) interpretation*. Here, acute impingement and entrainment are sufficient metrics, and are to be minimized without respect to cost considerations. The 316(b) choice is largely or entirely technology driven, based on the most literal interpretation of minimizing BTA. No special consideration is given either to distinguishing fish eggs and larvae from juveniles or adults, or distinguishing individual fish mortality from population impacts, or considering the role of confounding causal factors such as water temperature changes or overfishing on population health. AEI includes virtually any level of fish mortality caused by a CWIS. A version of this strategy has been proposed by the Riverkeeper.

- *Biocriteria or other proxy-based*. Here, a key tool is the use of quasi-scientific metrics, defined nationally, for concepts such as the littoral zone, biologically sensitive areas, aquatic zones of CWIS influence, or biocriteria-style metrics; the latter typically integrate a suite of ecological measures or professional judgments.[32] In contrast to some human health risks, a challenge here is to make such policy constructs work for ecological impacts varying widely in their nature, causes, amplitude, and significance. In August 2000, the EPA proposed a version of this strategy that includes standards dependent on water body type (river, estuary, marine, lakes) for new CWISs. This can be seen as a hybrid proposal combining a general value judgment on water body importance and a lower-level ecological risk assessment process using proxies.

- *Site-specific and risk-based*. Here, AEI is characterized on a site-specific basis with fish population (rather than acute) impacts being primary. AEI is evaluated in the context of both fisheries management and overall comparisons of stakeholder benefits and costs (Fig. 1). AEI defined in

> this way is close to a general definition of aquatic and stakeholder ecological risk, the primacy of population metrics being perhaps the only significant restriction. UWAG has proposed an approach like as an AEI definition, but its important features are that the characterization of AEI is site-specific and is limited to population impacts.[33]

While these are simplified positions, they capture key features of the approaches proposed by three major 316(b) stakeholders. The first option is the simplest pure technology standard and the last is the broadest site-specific risk-based approach. The first is forced to characterize AEI as any fish mortality because it is based on minimizing BTA, independent of cost or net benefits. In contrast, the last option provides a general framework for defining AEI on a case-by-case basis, and considers fish mortality adverse only when it has significant population consequences. In the second approach, a methodological compromise is attempted through general criteria tailored to water body types. The second approach is less clear about AEI than the other two, although AEI is implicit in the proposed proxy measures and selection of water body types. Thus, each policy approach implies something about AEI.

But in each case, the role of AEI either is not especially informative, or is just one part of a broader approach to 316(b) policy. To compare these 316(b) policy options then requires criteria not defined solely in terms of AEI. Implications of the three approaches can be briefly assessed as follows, using the organizing ideas above: CWA 316(b) policy vision, regulatory implementation, and knowledge base.

*"Strict" 316(b) interpretation.* First, the policy is relatively easy to implement; for example, the Riverkeeper has argued against the introduction of what they consider to be a needlessly complex assessment or stakeholder process. They have also effectively designated dry cooling, which minimizes evaporative water loss, as a generic minimizing BTA. An advantage of this particular technology rule is that it assures a high degree of fish protection and appears minimally subject to gaming or delay tactics in implementation (the latter being a Riverkeeper criticism of a site-specific or stakeholder approach).

In itself, a technology-based rule is neither good nor bad. Nearly all regulation is necessarily based on simplifications or aggregate rules needed to assure desired outcomes through implementable regulation. A regulated society has to accept some error tolerance between, on the one hand, an idealized best practice by cases, and, on the other, the implementation of uniform regulatory policy. The wastewater provisions of the CWA based on technology criteria, for example, show that such an approach can be done reasonably well. Indeed, as a matter of CWA interpretation, it seems fair to at least raise the issue of a possible analogy or disanalogy with this feature of CWA philosophy (though congressional financial support to repair the nation's once-dilapidated wastewater system is also relevant). It is also somewhat counterintuitive that the CWA should be used to improve water quality and protect aquatic life, but then be interpreted to allow large acute impacts to fish life through entrainment and impingement. So a “strict” 316(b) interpretation and its technology rule should not be dismissed immediately on methodological grounds, the problems of the 316(b) text notwithstanding.

But what is important is whether the *particular* uniform technology rule is a good one. Is the certainty it provides balanced or outweighed by other consequences, i.e., those representative of all stakeholder interests in Fig. 1? To what extent does the implied CWA policy vision represent a sound environmental choice? And how does the fish protection philosophy promoted as part of 316(b) cohere with what is known about how fish survive and reproduce, the different consequences of impingement and entrainment, and other resource management practices? A “strict” 316(b) approach is not necessarily in contradiction with a scientific perspective on ecosystems, but such a policy should account for these basic issues. A variant might be a “strict” 316(b) interpretation for CWISs or water bodies judged in need of the strongest protection. In that way, the CWA philosophy is preserved but targeted only to those conditions where it is clearly judged worthwhile. If there are *no* situations for which a uniform 316(b) technology standard is justified, then that would be useful to know as well. As another variant, UWAG has proposed a two-track option for new CWISs through which a conservative BTA choice could be implemented speedily, or a site-specific risk assessment could be carried out to better understand local impacts. This too partly preserves a CWA technology-forcing philosophy, but with greater flexibility.

*Biocriteria or proxy-based.* Biocriteria or other quasi-scientific metrics have several advantages: (1) a coarse technology standard is improved by measures more closely related to the outcomes of interest; (2) an approach to regulatory implementation is provided that is hopefully not as complex or practically overwhelming as more open-ended risk assessment processes; (3) a certain amount of predictability and fairness in implementation is provided; and (4) the rationale for various biocriteria or proxy guidelines might possibly be more easily justified on scientific grounds.

Biocriteria and regulatory proxies generally are motivated by the idea that the right criteria can become adequate regulatory tools. Constructed measures are often created on a site-specific basis for many environmental problems, so why not just do that at a general regulatory level? Of course, whether the right outcomes can be captured by the constructed measures is the great challenge. It is also predictable that if more cases and variability (multiple fish species, competing causal factors, water body management approaches, ease of modeling, etc.) needs to be captured, the less likely it is that the criteria will be successful. Given that such criteria are intended to have some kind of scientific base, they are open to criticism for being motivated by expediency rather than explanatory principles. The approach of creating regulatory proxies also has a long history in environmental regulation, and sometimes creates complex policy choices when hard value judgments are replaced by quasi-technical solutions.[34]

So the use of biocriteria or proxies has clear implementation advantages, and is an improvement over some pure technology rules for representing policy. But a difficulty is that important policy consequences may have to be reverse-engineered from the proxy measures because desired policy outcomes are only stated indirectly through the proxy measures. Therefore the intended policy vision may have to be backed out by seeing just what the criteria imply. The motivation to create an implementable policy is important, as is making pragmatic use of science. But ambiguity and lack of transparency in policy vision leaves the approach open to objections of confounding scientific tools and policy choices. A single point of agreement between the Riverkeeper and UWAG in their responses to the EPA's August 2000 proposed new facilities rule was their shared rejection of the EPA's proposed proxy measures (which were indeed discarded in the final November 2001 rule).

There is a great deal of experience in data collection, modeling, and interpretation from 316(b) experience, some of it overly complex or of limited decision-making value. For some situations, proxy measures or biocriteria may be useful, such as for managed reservoirs. But in general, it may be wiser to provide regulatory guidance for using recognized scientific approaches rather than designing new regulatory concepts whose scientific merits and operational value are yet to be established. One may ask regulators to exercise judgment in using the best knowledge available, rather than preprocessing that knowledge through a filter whose rigor has not been openly established. The EPA has organized their use of proxy measures for new facilities by water body type as a high-level means of prioritizing fish protection. That same overall approach could just as well be designed with water body guidance for assessment, modeling, and protection, instead of, or perhaps along with, proxy measures. Implementation is an important and often neglected policy objective, but must balance regulatory usefulness and scientific validity.

*Site-specific and risk-based*. This approach makes primary the value judgment that fish mortality is significant only when it has an effect on fish (or ecosystem) population health or viability, and not acute impingement and entrainment mortality by themselves. Attempts to characterize AEI by fixed population percentage losses or absolute numbers (e.g., biomass loss) appear largely to have failed, and technology rules or proxy measures are judged, from the site-specific perspective, to be conservative or inaccurate. Therefore, case-by-case risk assessment is proposed as the best means for more accurately and fairly defining AEI, and this perspective does reflect the local character of many ecological science studies.[35] The approach presumably includes local judgments about ecological, cost, and other tradeoffs, thus emphasizing roles for ecological (and social) value and technology effectiveness poorly addressed by technology or proxy-based approaches. The site-specific approach therefore has clear policy vision and is intended to make good use of the knowledge base. This policy vision however comes from contemporary risk assessment methods and is largely a substitute for the technology-based BTA perspective of the 316(b) text.

Proponents will note the traditional acceptance of probabilistic risk assessment methods for fisheries management of populations and not individual fish, and that for decades, dozens of 316(b) decisions have effectively been made on a site-specific basis. Other stakeholders, such as those favoring a "strict" 316(b) interpretation, will differ on whether those decisions were made correctly, or whether the fisheries perspective is appropriate.

Instead of proposing a substantive definition of AEI (i.e., one that does more than identify population effects), the site-specific approach instead proposes a process. Only broad advice is then also provided on how it is to be implemented in practice. For example, UWAG's AEI definition amounts to a compact definition of multiple values risk, in that each 316(b) decision is looked at as involving local tradeoffs among fish protection, 316(b) technology and energy production costs, and potential environmental side effects from alternative technologies. EPRI has recommended use of the EPA's own Ecological Risk Assessment guidelines as one approach (and it is odd that others should have to recommend the Agency's recommendations to itself). An important question about whatever process might be used is the nature of value judgments. UWAG, for example, while proposing a process inclusive of all stakeholder benefits and costs, does not indicate whether broad stakeholder participation is appropriate. Along with site-specific modeling or impact assessment, such a process may also entail a greater resource commitment than a uniform technology rule or a proxy-based approach. At the same time, more open-ended stakeholder-cum-risk-assessment processes are increasingly common, and 316(b) could be implemented using similar approaches.[36] The certainty achieved by the "strict 316(b)" approach for fish protection outcomes, or the uniformity of biocriteria or proxy rules, is here traded for a more open process. The latter is also just what the "strict" 316(b) approach may want to exclude because of a potential risk of underprotecting fish through process manipulation or delay.[37]

Finally, relevant to site-specific assessment is the value judgment of deciding on an appropriate dollar value for mitigation projects. It is often difficult to retrofit an existing CWIS to reduce entrainment at costs less than several tens or even hundreds of million dollars. In such situations, several 316(b) decisions have led to enhancement or mitigation projects including fish stocking or ecological improvements.[38] A key decision variable is the dollar amount to invest in the enhancement project, as there may be no obvious upper bound to how much should be spent. To create a 316(b) "nexus," some kind of ecological valuation may be used, but the uniqueness and, typically, nonmarketability of ecological resources makes ecological value itself highly site-specific and subject to stakeholder judgment. Lacking guidance or standards, the determination of a "just" enhancement budget can potentially become a negotiation exercise subject to a company's ability to pay, and therefore raises significant fairness questions for the CWIS owner.[39] Thus, site-specific AEI explicitly raises policy problems of ecological valuation, but ones which also have been implicitly conducted during all the years that 316(b) decisions have been made without explicit regulatory guidance.

**TABLE 1**
**Coarse Evaluation of Simplified 316(b) Policy Approaches**

| | CWA Policy Vision | Implementation | Knowledge Base |
|---|---|---|---|
| Strict 316(b) interpretation | Assure high protection by simplest technology rule with little regard for side effects or cost. Takes 316(b) at face value. | Straightforward (e.g., dry cooling or equivalent is BTA). | Emphasizes impingement and entrainment mortality. Population vs. individual distinction not too significant. |
| Proxy-based | Use specialized constructs to identify water bodies or populations for protection. Much 316(b) policy built into proxy constructs. | Straightforward, at least assuming constructs give meaningful results. | Compromise between policy definition and scientific concepts. |
| Site-specific and risk-based | Protect populations; aim for consistency with other fish management practices; otherwise assessments and tradeoffs are site-specific. Largely rewrites 316(b). | Open-ended, especially given ecological and modeling challenges. Effort-accuracy tradeoffs and stakeholder roles unclear. | In principle, makes best use of available science base, but process details unspecified. |

The three simplified policy proposals and their evaluations are summarized in Table 1. In summary, none of the three simplified 316(b) approaches should be faulted on principle, and indeed each has something to recommend. But neither does any completely answer natural problems raised in the areas of CWA vision, regulatory implementation, the science base, and the allocation of stakeholder benefits and costs. In any case, the evaluation of 316(b) policy proposals can be carried out through a simple functional model of important policy dimensions. With no "silver bullet," where does this leave the problem of developing 316(b) policy? A site-specific, risk-based approach comes the closest to the consequentialist, outcomes-based approach of Fig. 1. To the extent that 316(b) policy should reflect regulatory implementation concerns, or aspects of CWA philosophy deemed important, features of the other policy approaches may have to be incorporated into a hybrid policy.

That may be achieved by a more direct or inductive approach to setting 316(b) policy, meaning to more concretely identify undesirable environmental outcomes, as opposed to AEI generally, and to identify just what should happen through 316(b) regulation. For example, a 316(b) policy organized by water body types using proxy or technology rules helps ensure certain minimum levels of fish protection. If such an approach overshoots its goals for costs or benefits, it can, for example, be adjusted to allow for site-specific risk assessment under identified conditions, planning in a more integrated way for implementing policy for new vs. existing facilities, or ranking 316(b) problems as, for example, "eliminate," "do not repeat," "not significant," "possible concern," "monitor," "up to local judgment," etc. (All these may be addressed differently for new vs. existing facilities as well.) Regulation needs rules, but for 316(b) they need to either be sharply focused, so that they achieve desired outcomes without large error, or flexible, so that they can be adjusted in practice.

The challenge is to do both with sufficient certainty of achieving environmental progress while not consuming excessive regulatory or other social resources. That means being both focused and flexible, or combining and tailoring elements from different 316(b) proposals so that their various advantages are exploited without the shortcomings of each taken separately.

### THE U.S. EPA'S PROPOSED AND FINAL NEW FACILITIES RULES

This paper was written following publication of a Clean Water Act Section 316(b) *proposed* new CWIS facilities rule by U.S. EPA in August 2000, and was completed just as the *final* new facilities rule was published in the *Federal Register* in November 2001.[40] The August 2000 proposed rule motivated some of the paper's discussion, and the November 2001 final rule differs from the proposed rule in important respects. Publication of the final new facilities rule provided an opportunity to apply the framework proposed above by seeing how the final and proposed rules differ.

In the final new facilities rule, proxy definitions such as the controversial littoral zone concept and biocriteria are given up in favor of what is essentially a technology standard. The final rule applies to all water body types uniformly, unlike the proposed rule, which included different criteria for rivers, lakes, oceans, and estuaries. The main feature of the final new facilities rule is that CWIS capacity and flow (above certain minimums) should be no greater than that for a closed-cycle, but "wet" or evaporative (tower or pond), cooling system. Thus, BTA is not dictated, but CWIS protection should be equivalent to closed-cycle technology. The final new facilities rule also allows a CWIS permit applicant to carry out a site-specific study, very much like a "two-track" approach proposed by UWAG in response to the EPA's proposed new facilities rule. However, the level of fish protection, primarily impingement and entrainment reduction, still has to be equivalent to that provided by closed-cycle cooling. Thus, a site-specific risk assessment can only conclude that an alternative CWIS technology provides a level of protection greater or less than closed-cycle cooling, not that impingement or entrainment does or does not create AEI.

The following points summarize changes between the proposed and final rules using the comparative framework discussed above. A principal change was from a proxy-based to a technology-based approach, as just mentioned.

- AEI is again not formally defined, but almost. The EPA enumerates various adverse outcomes, including population changes, but the list is headed by impingement and entrainment. Thus the rule mainly provides protection against acute impingement and entrainment. The basic ecological value judgment appears to be that fish should be protected from large anthropogenic causes of mortality different from fishing. Impingement and entrainment are also considered important contributors to the cumulative risks faced by aquatic populations created by overfishing, nonpoint source runoff, and other hazards. The possibility of further characterizing AEI through the NPDES 316(b) process is largely foreclosed by limiting the relevant outcomes of site-specific risk assessment in a "two-track" process (see above) to those equivalent to closed-cycle cooling protection.
- The weakness of proxies, mainly the important littoral zone concept of the proposed rule, is acknowledged. Also missing from the final rule is differential protection based on water body types. Instead, an equivalent technology rule is considered appropriate for 316(b) along the lines of CWA philosophy generally. Thus the precise nature of the fish protection intended is somewhat less clear.
- The stakeholder value hierarchy of Fig. 1 is reflected in the EPA's consideration of cost and other environmental outcomes. Based on their economic analysis, the EPA found that, because only a small number of future plants were expected to now *not* use once-through cooling, and because closed-cycle technology is already widely used, economic impacts on new facilities were justified by the fish protection provided. Additional emissions associated with closed-cycle cooling and the aesthetic impacts of cooling towers or ponds were similarly judged acceptable tradeoffs. Economic analysis also led the EPA to favor wet vs. dry cooling, the latter having been supported by the Riverkeeper as BTA. It appears that while the EPA uses very approximate fish protection estimates, the cost tradeoffs are sufficiently small on a national level for the limited accuracy to be unproblematic. Setting the cost boundary below that for dry cooling may have cost implications for the forthcoming existing facilities rules. It could of course also be argued that the EPA's estimates of benefits and costs do not justify the tradeoffs implicit in the new rule.
- As indicated already, BTA (for intakes above certain threshold flows) is defined by comparison with wet closed-cycle cooling. Since AEI is not defined, that implies that the choice of minimizing BTA is a key piece of the 316(b) nexus. An important aspect of this definition is that the EPA does not identify particular technologies: "EPA emphasizes that it is not requiring wet cooling, but that it is establishing performance-based technology requirements on the dynamic flow of the cooling water intake structure that reduce impingement and entrainment at a level that is achieved by using closed-cycle cooling"[4, p. 263]. The use of a technology equivalent may also have implications for forthcoming existing facilities rules. Again, EPA

appears to have made traditional CWA (wastewater) philosophy central to 316(b).

- Regulatory implementation is identified multiple times by the EPA as a concern for site-specific approaches that require the use of mathematical models. The complexities of fish population assessment and modeling, including the need for multiyear studies, and the problems in characterizing CWIS causality appear to have been major reasons that the EPA rejected risk assessment approaches and instead designed a technology standard.[41] Evidence provided by the EPA justifying the new facilities rule included studies on intake-flow velocity and (possibly dated) impingement and entrainment counts from various 316(b) studies. The EPA also states that forthcoming 316(b) rules for existing CWISs may be entirely different, with no precedent set by the new facilities rule.
- Fisheries management approaches, including the concept of maximum sustainable yield (MSY), appear to be rejected by the EPA as a paradigm relevant to 316(b). That is, they do not consider impingement and entrainment a resource harvest to be managed like fishing. Further, the problem of collapsed fish stocks is cited as reflecting the need for fish protection, with CWISs identified as a contributing factor along with nonpoint source runoff and other pollution. The EPA appears to be upholding a perspective that fish may be managed in one way as a fisheries resource, and in another way as in need of protection. The recognized differences between adult fish mortality and that for eggs and larvae plays little or no role in the EPA's rule, but likely because there is no useful technology standard which differentiates the two, not because it is scientifically unimportant.

These comparisons show that the comparative approach developed in this paper is broadly useful. The issues differentiating the proposed and final rules were anticipated by the framework, though of course it could not be predicted which way policy options might go. For example, the EPA might also have incorporated elements of a site-specific and risk-based approach, rather than relying on a pure technology standard. The EPA can be argued to have made an improvement over its previous proxy-based proposed rule without substituting some weaknesses of the "strict" 316(b) interpretation used above. The absence of any proposed implementation philosophy for site-specific risk assessment, including the need to simplify and interpret modeling studies, appeared a strong reason to revert to a technology standard; that still implies that EPA choose here not to follow their own ecological risk assessment guidelines and, more broadly, approaches to risk assessment and environmental policy emphasizing tradeoffs among social values . The comparative process may provide further insight into policy options as debate evolves over rules for existing facilities.

## APPENDIX: SUMMARY OF RESPONSES TO U.S. EPA'S AUGUST 2000 PROPOSED NEW FACILITY RULE

### Simple Comparisons Between the Riverkeeper's and Utility Water Act Group's Responses to EPA's August 2000 Proposed New Facilities Rule[1,3]

| Issue | UWAG[8] | Riverkeeper[9] |
|---|---|---|
| Motivation of CWA | Emphasis on legalistic aspects of CWA interpretation; BTA a confusion; cost is relevant as expressed elsewhere in CWA. | Fairness in protections; aim for best technology; straightforward interpretation of CWA philosophy and 316(b). |
| AEI as focus | Correct approach; needed to make sense of what is “minimized” in 316(b) and identification of BTA; should be defined for a site; is the part of 316(b) that relates to actual outcomes; lack of AEI definition is central focus of critique. | Any impact counts. Do not need to finesse. |
| Site-specific study | Says is scientific approach. AEI defined by site-specific study. | Would be co-opted; overly complex; impossible to be fair. |
| Population impacts vs. direct counts | Population is the only relevant impact, except for endangered species. | Is included in value judgment that individual fish impact defines harm. |
| Impingement vs. entrainment | Might separate value judgments for adult fish and eggs/larvae. | No separation of roles of larvae and adult fish. |
| BTA as focus | Error to take technology perspective, as opposed to outcomes. CWA fundamentally ill-conceived here. | Correct approach, consistent with CWA philosophy for waste and stormwater. |
| Fisheries comparison | Relevant. Need consistent resource management policies for fisheries and other consumptive uses. | No comment made. |
| Compensation | Relevant in that populations | Bogus use to explain away |

| | | |
|---|---|---|
| or density-dependence | can be healthy with significant acute mortality. | impacts. |
| Cost | Should use cost/benefit criteria. Implicit elsewhere in CWA. | Not relevant; not a concern as is low for new plants; not part of 316(b). |
| Role for new vs. existing plants | Unclear. | Unclear. |
| History of 316(b) decisions | No serious ecological problems. | Several bad decisions made. |
| EPA use of water body type, littoral zone, etc. | Opposed to EPA definitions, but categories are ones that would be used for site-specific analysis. | Also opposed; seen as a diversion from technology standard. |
| Volumetric analysis | No basis for use as a proxy. | Key goal: leads to dry cooling; is seen as obvious influencing factor. |
| Flow limits | Against. | Not strong enough. |
| What is a CWIS? | Excludes other parts of system such as cooling towers. | Includes system all the way to cooling towers. |
| Technology basis | Opposes. | Supports and sees as consistent with CWA. |
| Risk-based approach | Desired as in fisheries management. | No comment made. |
| Stakeholder role | Implied, not explicit in proposal. | Too complex to implement. |
| Population modeling | Required. | Too complex. |
| Other environmental outcomes | Relevant, and should be included in cost/benefit criteria. | Not significant impacts, e.g., noting existing dry cooling practices. |
| Mitigation | Relevant. | Only for past harm. |
| Similarity to thermal | Similar in that effects differ from chemical pollution risks. | Not applicable. |

## NOTES

1 See [1, chapter 11] for a summary of impingement and entrainment data across various water body types, fish species, and CWIS facilities. About 1,000 steam-electric power plants are affected by 316(b), and approximately 70 trillion gallons of water per year are withdrawn by power plants and other facilities for cooling purposes; nearly all of that water is a nonconsumptive use, i.e., it is returned, warmed, to the source body. The principal events in the history of Section 316(b) include:

- 1972: The Federal Water Pollution Control Act Amendments become law including Section 316(b).
- 1973: EPA publishes a “Development Document” addressing 316(b).
- 1976: EPA publishes final 316(b) rules.
- 1977: 316(b) rules are remanded back to the EPA as the result of an electric utility industry lawsuit.
- 1979: EPA withdraws its 316(b) regulations (after this time, the 1973 Development Document is used widely for guidance in making 316(b) decisions).

- 1993: Several environmental groups, led by the Hudson Riverkeeper organization, bring suit against EPA in *Cronin v. Browner* to force EPA to issue regulations (John Cronin is a founder of the original Hudson Riverkeeper[2]).
- 1995: A consent decree in *Cronin v. Browner* provides a schedule for issuing regulations
- August 2000: EPA publishes *proposed* new CWIS facility rules[1,3].
- November 2001: EPA publishes *final* new CWIS facility rules[4]; rules for existing CWIS facilities are scheduled for 2002–2003, and may depend on the volume of water processed by the CWIS.

See Anderson and Gotting[5] and May and van Rossum[6] for 316(b) history and Dixon et al.[7] for perspectives on 316(b) policy and science. An important observation, made often by the electric power industry and noted in the text, is that 316(b) decisions have been made *de facto* on a case-by-case, site-specific basis for decades; critics claim that many CWISs currently impinge or entrain more fish than is ecologically acceptable, while the industry claims zero or de minimus ecological impacts over decades of once-through CWIS usage.

2 See the Utility Water Act Group[8] and Riverkeeper[9] for responses to the EPA's August 2000 proposed new facilities rule[3].

3 "Adverse environmental impact is a reduction in one or more representative indicator species that (1) creates an unacceptable risk to the population's ability to sustain itself, to support reasonably anticipated commercial or recreational harvests, or to perform its normal ecological function, and (2) is attributable to the operation of the cooling water intake structure. Because this definition is drawn from scientific principles fundamental to natural resource management, it can be interpreted using the same concepts and analytical techniques used by fisheries scientists and resource managers…. The proposed definition turns on 'unacceptable risk.' What risk is 'unacceptable' is a function of a number of biological and social factors, which must be managed through a scientific risk assessment and risk management process"[8]. This definition leaves open the role for a stakeholder process to characterize acceptable risk and its tradeoffs, and the modeling or data collection simplifications that may be needed for a practical regulatory process. These implementation issues noticed by the EPA and the Riverkeeper are made part of the evaluative framework in below.

4 Terrestrial impacts are included by Anderson and Gotting[5], but not UWAG[8], but this seems an insignificant issue. For a rare fisheries example of an aquatic-terrestrial impact, see McEvoy[10].

5 General comparisons of benefits and costs have been suggested[5,6,8]. These authors do not refer to well-known methods of multiple values (or multiple stakeholder) decision analysis as in Dunning et al.[11], Gregory et al.[12], and Keeney[13]. The value hierarchy of Fig. 1 provides the foundation for a multiple values and stakeholder approach to 316(b) decisions; McDaniels et al.[14] use this type of high-level analysis to structure an environmental decision without complete quantification of measures, which could also be useful for many 316(b) decisions. The EPA's final new facilities rule[4] discusses the tradeoffs implied by Fig. 1, but does not endorse a stakeholder approach. The comparative policy framework put forward below identifies some 316(b) issues that complicate a values-focused methodology[13], and is "prescriptive," meaning it combines descriptive and normative views of decision making[15].

6 UWAG's proposal[8] is to make this case-by-case process more explicit by following general risk assessment guidelines. Thus their point that some AEI role is needed is valid. UWAG's contention that ecological impacts are inherently site-specific is supported by the National Research Council[16].

7 Defining BTA as dry cooling is a major part of the Riverkeeper proposal[9].

8 That is, tradeoffs may be more or less formal, ranging from heuristic judgments of an expert panel[17] to the use of codified dollar-equivalents based on local fisheries rules[18,19,20]. These judgments may become quite relevant as rules for existing facilities are debated. As mentioned in the text below, 316(b) ecological valuation may be hard to disentangle from stakeholder negotiations involving the determination of a mitigation or enhancement settlement amount.

9 This is similar to elements of the UWAG proposal and shows how close it is to the multiple values approach of Fig. 1; see also note 5 above.

[10] For an example of adverse population decline, see Ambrose et al.[21]: "Besides entraining large numbers of larval and juvenile fish, SONGS [San Onofre Nuclear Generating Station] kills 21 to 56 tons of larger fish each year when they are impinged on the screens in the intakes of Units 2 and 3. The impingement loss itself was not considered by the MRC [Marine Review Committee] to be a substantial effect. However, this loss contributes to the significant decline in local midwater fish abundances detected by the MRC, although other factors (such as the local increase in turbidity, which might cause fish to leave the area) also appear to be involved. The MRC considered the reductions in local fish populations to be a substantial, but local, impact." For an example involving ecosystem health at lower trophic levels, see the California Energy Commission[17]. At the same time, see Rose[22]: "Examples of overfishing causing population declines are numerous. However, despite extensive efforts, definitive quantitative demonstrations of dramatic fish population declines (especially for coastal species) caused by anthropogenic changes in EQ [abiotic aspects of environmental quality, including entrainment and impingement] are embarrassingly lacking…. As the stakes continue to rise, either we must show the importance of anthropogenic changes in EQ to fish population dynamics, or we must conclude that these changes are insignificant relative to other sources of variation."

[11] See Dixon et al.[7] for several approaches to biocriteria and perspectives on their application.

[12] Several of the EPA's proposals for new (vs. existing) CWISs fall under this category[3].

[13] For example, at the Chesapeake Bay Chalk Point plant[18], several proposed hypotheses and tests about tidal flows and larval transport in the Patuxent River were needed to ascertain that the key consequence of anchovy entrainment was forage fish reduction. The complexity and cost of determining this outcome, and its relative insignificance, may be compared to the much more direct task of reducing impingement from several millions of fish annually to less than one-half million through successful installation of a barrier net. Historical studies such as these may become valuable input for developing guidelines for existing facilities rules. The importance of hypothesis formation and "confrontations" with data in ecological analysis is discussed in Hilborn and Mangel[23].

[14] A good example of population modeling for the Ohio River using a basic Leslie matrix approach is in Lohner et al.[24], in which a largely uniform modeling and data collection approach is used to model multiple species at several independent river pools, as opposed to modeling each pool using a different approach. For a contrasting example of intensive, site-specific modeling, see Lorda et al.[25]. The latter is also an example in which stochastic modeling is used to argue for a relatively small role for entrainment, compared to regional overfishing, as a cause of local winter flounder population decline.

[15] For an example, see the California Energy Commission[17], in which larval and egg depletions were equated to wetland acreage, which in turn was cost out as a restoration program, and then used to set a final dollar valuation. However, the final mitigation/enhancement choice involved land easements and control of nonpoint source runoff, but not wetland development; the former were judged the higher priorities for improving ecological health at Elkhorn Slough. By putting 316(b) in an overall context of water body management, the CEC process in this way illustrates a desirable feature of site-specific valuation and decision making.

[16] For an example for which declines in a single local winter flounder species (Niantic River, Connecticut, stock) becomes a special focus, see Lorda et al.[25]

[17] Lohner et al.[24] is an example in which the aggregation from stochastic process to outputs more useful to decision making is carried out in some detail. On the important role played by modelers in making science useful for policy see Jasanoff[26] and van Winkle and Kadvany[27].

[18] For an example in which fisheries valuation approaches were utilized, see Bailey et al.[18] For a site-specific approach involving lower trophic levels, see California Energy Commission[17]. For additional cost examples, see May and van Rossum[6]. Nonmarket ecological valuation is not nearly as difficult as many seem to believe, but does depend on recognizing the role to be played by a stakeholder process and the constructive, or stakeholder-defined, nature of valuation judgments[11,12]. For empirical studies relevant to environmental measurement and value definitions see Irwin et al.[28] and Fischhoff[29].

19 On EPA's ecological risk assessment process see [31, 32]. UWAG comes closest to addressing process issues through an AEI definition entailing comparisons of all relevant stakeholder benefits and costs, though it is unclear whether UWAG includes that too as part of a scientific risk assessment process, which it is not. On the need for science-stakeholder interactions, see the National Research Council[30].

20 This issue appears not to be addressed in any 316(b) proposal, but see also Rose[22], p. 381: "Without modeling approaches, isolating EQ [abiotic environmental quality] effects on long-lived species, such as fish, requires years of monitoring that span a range of natural conditions. Often, sufficient data to detect effects are not obtained until it is too late and the population has dramatically declined or easy recovery is hindered. This is not to imply that data collection is not needed. The accuracy and precision of model predictions depends on the quality and quantity of the empirical data used to develop and corroborate the model. But maintaining funding for sufficient long-term monitoring and waiting decades for definitive signals in the data is not possible in many situations…. While population-level analyses tend to require fewer data that are more readily available, they also imply several simplifying assumptions that are often briefly stated and then ignored…[and] the effects of community-level interactions are too often dismissed early in analyses without consideration of how they would affect predicted population responses."

21 In the terminology of the EPA's Ecological Risk Assessment process[31,32], these measurement criteria define risk assessment endpoints.

22 See Adler et al.[33]. The terms "entrainment" and "impingement" do not appear in the book's index.

23 One meaning is that fish can be managed as a resource harvest for CWISs just as they are for fisheries. That view can certainly be defended, but two issues have to be addressed. First is that one can have a division of regulatory labor in which one agency protects and another sets fishing levels, assuming there are not absurd inconsistencies between the two. Depending on the water bodies involved, the health of the fisheries, and other environmental protection goals, it is not immediately obvious that a uniform "resource harvest" approach is necessarily correct. Second, compared to a straightforward "counting" of impacts, as a society we have biases about how we account for all kinds of risks, with a great lack of mathematical consistency. While such unreflective views can be inconsistent, they are not necessarily wrong, as they may be ways of expressing complex, multidimensional attitudes toward risk[34,35], combined with legal and other institutional constraints. Such views can certainly be criticized and changed, and attitudes toward fish protection in different contexts may need that. But then the resource harvest view might be combined with a kind of risk communication or broader policy campaign, and should not be seen as obviously right or wrong[36].

24 See Singer[37], who places fish just inside the phylogenetic boundary of animals able to suffer pain, and argues therefore against their use as a human resource. The argument would not, however, obviously extend to entrainment of eggs and larvae. Singer, however, is also a complete consequentialist who would not distinguish fish killed for food production from those killed to produce electric power.

25 Fish population models are often probabilistic, but the scientific basis for which stochastic model is correct and how to estimate parameters or design a model (fecundity and survival rates, habitat resource constraints, environmental factors, whether compensation is appropriate, etc.) can be subject still to considerable uncertainty. On challenges for satisfactorily establishing population declines in the fisheries industry, see McEvoy[10].

26 The comparison may be a bit strong, given that literal enforcement of the 1958 Delaney Clause could actually *increase* cancer risks[38]. Other differences are that 316(b) deals only with ecological outcomes, and "minimizes" BTA with respect to an undefined AEI, while the Delaney clause implicitly defines adverse as within the limits of detection.

27 The effort-accuracy tradeoff[39] is an important concept in behavioral decision theory that is useful for understanding the practical requirements of policy analysis. As an illustration, and as indicated below, the EPA made regulatory implementation (specifically with respect to the NPDES permitting process) an important factor in their final rule-making. This behavioral consideration is a main difference between a pure values-focused approach[13] and the evaluative framework proposed in the text. Keeney[13] argues against "alternatives-focused" decision making, which would include technology-based rules defined independently of their consequences. However, in creating regulations, due to the quality of scientific knowledge available and the resources and priorities for regulatory implementation, rule-makers may justifiably resort to alternative-based strategies.

28 Anderson and Gotting[5] correctly criticize the open-endedness of the current 316(b) case-by-case process, but they do not appreciate that their site-specific approach does little to limit the range of normal variability known in empirical and analytical studies of multiple values decision making; see the decision analysis references in note 5 above.

29 For a range of mitigation values see May and van Rossum[6].

30 Anderson and Gotting[5] argue that it is a scientific judgment that the population level is correct. But this view is mistaken: this is a value judgement, held by some scientists, perhaps not by others. It is not a consequence of any scientific theory, in contrast to whether one can easily influence a population by managing early life stages, or the relevance of high levels of natural mortality for eggs and larvae. Arguments that all ecological measures are value-laden by some implicit or explicit choices for ecological health or well-being are in Lackey[40,41] and Shrader-Frechette and McCoy[42].

31 An unfortunate simplification in this paper is not better distinguishing existing and new facilities. Indeed, the whole 316(b) debate is complicated by the sequential promulgation of rules (first new, then existing), making for considerable second-guessing and possible miscommunication about the consequences of earlier rules for later ones. For example, existing facility rules are to be further broken down by water volumes, with that measure likely a key proxy for the largest, often nuclear, plants. In their final new facilities rule[4], the EPA also states that a completely different approach is possible for existing facilities. Given the large cost of retrofitting existing CWIS to reduce entrainment, their approach to existing facilities will likely be quite different than for new ones.

32 See Dixon et al.[7] for papers on biocriteria approaches, strengths, and weaknesses. The construction of such quasi-scientific metrics occurs elsewhere in environmental policy, such as for pesticides or contaminated site regulatory ranking. A key problem with such metrics is that the final "units," while often providing useful diagnostic information, have no clearly defined dimensions for use as significance measures.

33 See note 3 above for the UWAG definition.

34 See Landy et al.[43] on substituting technical problems for value choices. Both the Riverkeeper and UWAG have criticized some of the EPA's biocriteria proposals, perhaps the single point on which they agree. But these groups may not recognize the need for such simplifications in regulatory policy, and why such measures appear for many environmental decisions such as those involving contaminated waste sites and pesticide safety (see note 27 above). Proxy measures are one approach to making use of science in a regulatory setting, but another approach is to allow for more flexible judgmental process of scientific outputs, and not to try to "customize" science to policy using quasi-scientific constructs. The EPA's final new facilities rule[4] rejects the biocriteria and proxies approach.

35 See National Research Council[16] on the ecological method.

36 See note 5 above.

37 This concern is raised by Riverkeeper[9] and may reappear in debates about forthcoming existing facilities rules.

38 See May and van Rossum[6], California Energy Commission[17], and Bailey[18] for various mitigation or enhancement projects. Some mitigation examples may indicate that the large project budgets involved, and modest benefits achieved, leave room for improvement[6].

39 For relevant issues, see Zajac[44].

40 The EPA's *proposed* new facilities rule is USEPA 2000[3] and the *final* new facilities rule is USEPA[4].

41 See Foster and Huber[45] for the scientific, judgmental and legal difficulties in identifying causality; for a 316(b) example involving over-fishing on Long Island Sound, see Lorda et al.[24].
